# Retraction Note: Senescent immune cells accumulation promotes brown adipose tissue dysfunction during aging

**DOI:** 10.1038/s41467-026-79227-9

**Published:** 2026-11-09

**Authors:** Xu Feng, Liwen Wang, Ruoyu Zhou, Rui Zhou, Linyun Chen, Hui Peng, Yan Huang, Qi Guo, Xianghang Luo, Haiyan Zhou

**Affiliations:** 1 Department of Endocrinology, Endocrinology Research Center, Xiangya Hospital of Central South University, 410008 Changsha, Hunan China; 2 National Clinical Research Center for Geriatric Disorders, Xiangya Hospital, 410008 Changsha, Hunan China; 3 Key Laboratory of Organ Injury, Aging and Regenerative Medicine of Hunan Province, 410008 Changsha, Hunan China

Retraction Note to: *Nature Communications* https://doi.org/10.1038/s41467-023-38842-6, published online 02 June 2023

The Editors have retracted this article. Following publication, concerns were raised regarding the accuracy of data reported in several figures and source data files. During the subsequent investigation, the authors identified multiple errors affecting figures, source data, statistical annotations and figure labelling.

The identified issues include duplication of source data values in Fig. 5d, incorrect values reported in Fig. S6g, data-entry errors affecting Fig. 7g and Fig. S3a, an incorrect value included in the source data for Fig. 6b, incorrect assignment of condition labels in Fig. 5e, incorrect statistical comparison labels in Figs. 2e-g and Fig. S5f, and incorrect figure labelling in Fig. 4j.

The authors provided replacement data and explanations for these issues. However, during the journal’s assessment, it was not possible to satisfactorily determine how the published values in Fig. S6g had been generated. The explanation provided by the authors did not fully account for all affected values, and the original native qPCR files required to independently reconstruct the workflow were no longer available.

Given the number and nature of the errors identified across the article and the inability to fully establish the provenance of certain published values, the Editors no longer have confidence in the integrity of the data reported in this Article.

Authors Hui Peng, Yan Huang and Haiyan Zhou agree with this retraction. Author Xu Feng agrees to the retraction but not to the retraction wording. The remaining authors have not responded to correspondence regarding this retraction.

